# Adenosine A_3_ receptor elicits chemoresistance mediated by multiple resistance-associated protein-1 in human glioblastoma stem-like cells

**DOI:** 10.18632/oncotarget.12033

**Published:** 2016-09-15

**Authors:** Angelo Torres, Yosselyn Vargas, Daniel Uribe, Catherine Jaramillo, Alejandra Gleisner, Flavio Salazar-Onfray, Mercedes N. López, Rómulo Melo, Carlos Oyarzún, Rody San Martín, Claudia Quezada

**Affiliations:** ^1^ Laboratorio de Patología Molecular, Instituto de Bioquímica y Microbiología, Facultad de Ciencias, Universidad Austral de Chile, Valdivia, Chile; ^2^ Instituto Milenio de Inmunología e Inmunoterapia, Facultad de Medicina, Universidad de Chile, Santiago, Chile; ^3^ Servicio de Neurocirugía, Instituto de Neurocirugía Dr. Asenjo, Santiago, Chile

**Keywords:** adenosine receptors, ATP-binding cassette (ABC) transporter superfamily, glioblastoma stem-like cells, multiple drug resistance

## Abstract

MRP1 transporter correlates positively with glioma malignancy and the Multiple Drug Resistance (MDR) phenotype in Glioblastoma Multiforme (GBM). Evidence shows that the MRP1 transporter is controlled by the adenosine signalling axis. The aim of this study was to identify the role of adenosine on the MDR phenotype in Glioblastoma Stem-like Cells (GSCs), the cell population responsible for the tumorigenic and chemoresistance capabilities of this tumour. We found that GSCs have increased intrinsic capacity to generate extracellular adenosine, thus controlling MRP1 transporter expression and activity via activation of the adenosine A_3_ receptor (A_3_AR). We showed PI3K/Akt and MEK/ERK1/2 signaling pathways downstream A_3_AR to control MRP1 in GSCs. *In vitro* pharmacological blockade of A_3_AR had a chemosensitizing effect, enhancing the actions of antitumour drugs and decreasing cell viability and proliferation of GSCs. In addition, we produced an *in vivo* xenograft model by subcutaneous inoculation of human GSCs in NOD/SCID-IL2Rg null mice. Pharmacological blockade of A_3_AR generated a chemosensitizing effect, enhancing the effectiveness of the MRP1 transporter substrate, vincristine, reducing tumour size and the levels of CD44 and Nestin stem cell markers as well as the Ki-67 proliferation indicator. In conclusion, we demonstrated the chemosensitizing effect of A_3_AR blockade on GSCs.

## INTRODUCTION

Glioblastoma Multiforme (GBM), classified as a grade IV astrocytoma by the World Health Organization (WHO), is considered the most common and aggressive tumour of the Central Nervous System (CNS) [1, 2]. Standard management for GBM patients involves surgical resection of the tumour, followed by radiation and chemotherapy with temozolomide (TMZ) which results in a survival rate up to 15 months [3]. Anti-angiogenic agents, and more recently immunotherapeutic approaches, are being developed to improve GBM prognostics [3, 4]. However, since it is a highly infiltrative tumour, cancer cells often invade healthy brain tissue and evade surgical resection which inevitably leads to early reoccurrence [5]. In addition, the intrinsic properties of tumour cell populations produce poor outcomes in almost all evaluated therapeutic alternatives.

A key features of GBM is its high chemoresistance to a broad spectrum of antitumor drugs, a phenomenon known as Multiple Drug Resistance (MDR) [6]. Acquisition of the MDR phenotype correlates with overexpression of members from the ATP-binding cassette (ABC) transporter superfamily [7]. These transporters are efflux pumps that translocate a wide range of substrates, such as lipophilic and xenobiotic molecules, to the extracellular environment using energy from ATP hydrolysis [7]. Analyses of biopsies and GBM cell lines, T98G and G44, revealed that the Multiple drug Resistance-associated Protein-1 (MRP1) is the predominant ABC transporter in GBM, compared to other transporters with roles in other types of cancer such as ABCG2 (also known as BCRP or Breast Cancer Resistance Protein) and P-glycoprotein (P-gp) [8]. Inhibition of MRP1 activity increases cell sensitivity to cytotoxic and antiproliferative effects of antineoplastic drugs such as vincristine, etoposide and taxol [8], representing a possible therapeutic target to control GBM reoccurrence.

GBM shows remarkable intratumour heterogeneity and cell hierarchy, not only at the morphological level but also in regards to cell differentiation status [9, 10]. Within the tumour a cell subpopulation called Glioblastoma Stem-like Cells (GSCs), which share some intrinsic properties with normal stem cells, such as self-renewal, proliferation and multi lineage differentiation [11, 12], are more resistant to radiation and chemotherapy than normal progenitor cells and differentiated tumour cells [12, 13]. This cell subpopulation gives rise to the tumour mass and cell heterogeneity and is therefore may be responsible for GBM reoccurrence following the use of chemotherapeutic agents [14, 15]. However, the mechanisms that induce the MDR phenotype remain practically unexplored. Some studies have detected expression of MDR transporters, particularly MRP1, MRP3, ABCG2 and P-gp in GBM [16–19]. Jin and col., observed a significant increase in MRP1 expression levels after exposition to etoposide, suggesting that this ABC transporter induces an adaptive response [20].

Inhibition of MRP1 extrusion activity is of clinical relevance in counteracting GSCs mediated reoccurrence. Therefore, understanding the mechanisms that control this transporter has become a hot topic. We have previously identified a relationship between expression and activity levels of ecto-5′-nucleotidase (CD73), an enzyme that catalyses AMP dephosphorylation to adenosine, with the MDR phenotype in glioblastoma cells [5]. This suggests a link between intratumour environment and MDR phenotype also exists. Adenosine is increased in tumour hypoxic medium [21] and its signalling through P_1_ receptor family members may mediate important responses that enable tumour growth. Indeed, it is well known that adenosine mediates tumour immunosuppression [22]. The aim of this work was to study the effects of adenosine on the MDR phenotype in GSCs. We also explored pharmacological intervention of adenosine receptor signalling to chemosensitize GSCs to conventional anti-cancer agents.

## RESULTS

### Glioblastoma stem-like cells have an intrinsically increased capability to generate extracellular adenosine

When maintained in serum-free neurobasal medium, human GBM Primary Culture (PC) cells and the human U87MG cell line were capable of forming non-adherent cell clusters or neurospheres, a cell subpopulation enriched in Glioblastoma Stem-like Cells (GSCs) (Supplementary Figure S1). Immunocytochemistry staining of GSCs derived from U87MG and PC cells showed expression of Stem Cell markers CD44 and Nestin (Supplementary Figure S1A). When GSCs of the GBM PC and U87MG cell line were cultured in differentiating medium containing foetal bovine serum they grew as an adherent monolayer culture and expressed differentiation markers of neuronal and glial cell, TUBB3 and GFAP, respectively (Supplementary Figure S1A). PC and U87MG neurospheres had a high percentage of cells positive for Stem Cell markers (Nestin and CD133), compared to adherent cells (Supplementary Figure S1B), analyzed by flow cytometry. Moreover, we also observed a higher mRNA expression level of Stem Cell markers (CD44, Nestin and CD133) in neurospheres than adherent U87MG cells (Supplementary Figure S1C). After U87GM-neurospheres were exposed to medium containing serum for 5 days we measured the kinetics of Stem Cells markers (Nestin, CD133 and CD44) and found that these markers disappeared in a time-dependent manner (Supplementary Figure S1D). In contrast, differentiation markers (TUBB3 and GFAP) increased their expression under these conditions (Supplementary Figure S1D). These results demonstrate the stemness abilities of our GSCs in neurosphere cultures derived from U87MG and PC cells.

**Figure 1:**
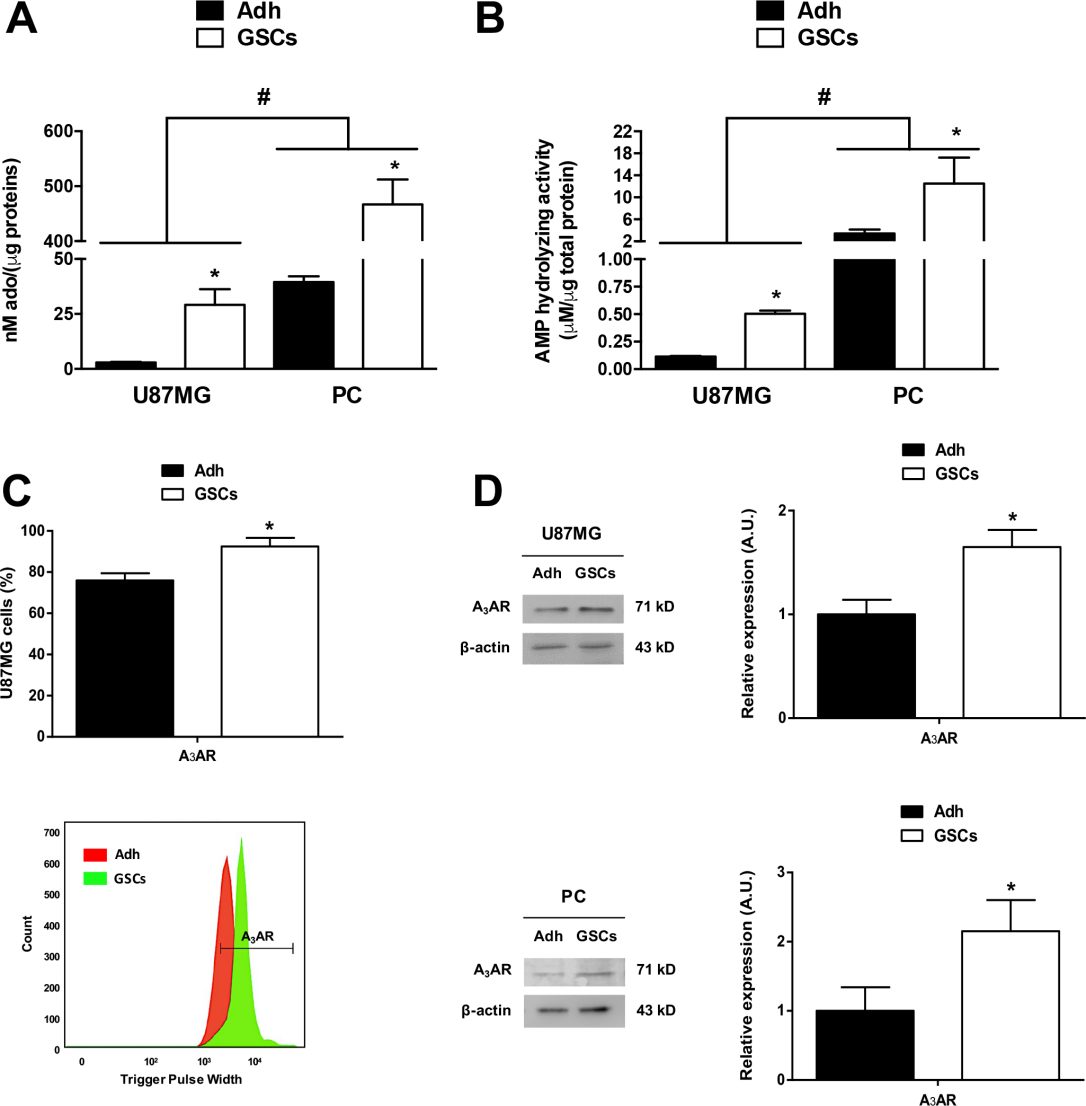
Adenosine metabolism in glioblastoma stem-like cells Extracellular adenosine accumulation and AMPase activity in U87MG and PC of adherent glioblastoma cells (Adh) and their respective GSCs, measured by HPLC. (**A**) Measurements of extracellular adenosine accumulation in U87MG and PC Adh cells and GSCs at day seven of culture. (**B**) AMPase activity in U87MG and PC of Adh cells and GSCs exposed to AMP (400 nM) for 15 min in Tyrode's buffer at 37°C and controlled oxygen levels. Adenosine concentrations in A) and B) were normalized to the total protein concentration in each test. (**C**) Flow cytometry graph of A_3_AR on U87MG Adh cells and GSCs at day seven of culture (upper panel). Representative flow cytometry histogram is present (lower panel) (**D**) Western blot of A_3_AR on U87MG and PC Adh cells and GSCs at day seven of culture. Graphs represent the mean ± S.D. *P < 0.05 Adh versus GSCs; ^#^*P* < 0.05 U87MG versus PC. *n* = 6.

The Multiple Drugs Resistant (MDR) phenotype has been linked to purinergic signalling in adherent population cells (also called differentiated cells) from glioblastoma [5]; however, the role of adenosine signalling in GSCs has not been explored. Our first approach was to quantify extracellular levels of the adenosine nucleoside in glioblastoma cells. We found a ten-fold y a thirteen-fold increase of extracellular adenosine in U87MG and PC GSCs respectively, compared to their differentiated cells (Figure 1A). AMPase activity was also higher in U87MG and PC GSCs compared to differentiated cells (Figure 1B), suggesting that purinergic signalling is essential to GSCs physiology. We previously observed 5′-ectonucleotidase (CD73) and adenosine A_3_ receptor subtype (A_3_AR) overexpression in GBM specimens compared to peritumoral tissues [5], but cell type specific distribution has not been evaluated. Via flow cytometry we observed that A_3_AR was present in more than 90% of GSCs (Figure 1C); similarly, receptor protein content was higher in U87MG and PC GSCs compared to their adherent cells (Figure 1D).

### The adenosine A_3_ receptor increases MRP1 transporter expression and activity in GSCs

**Figure 2:**
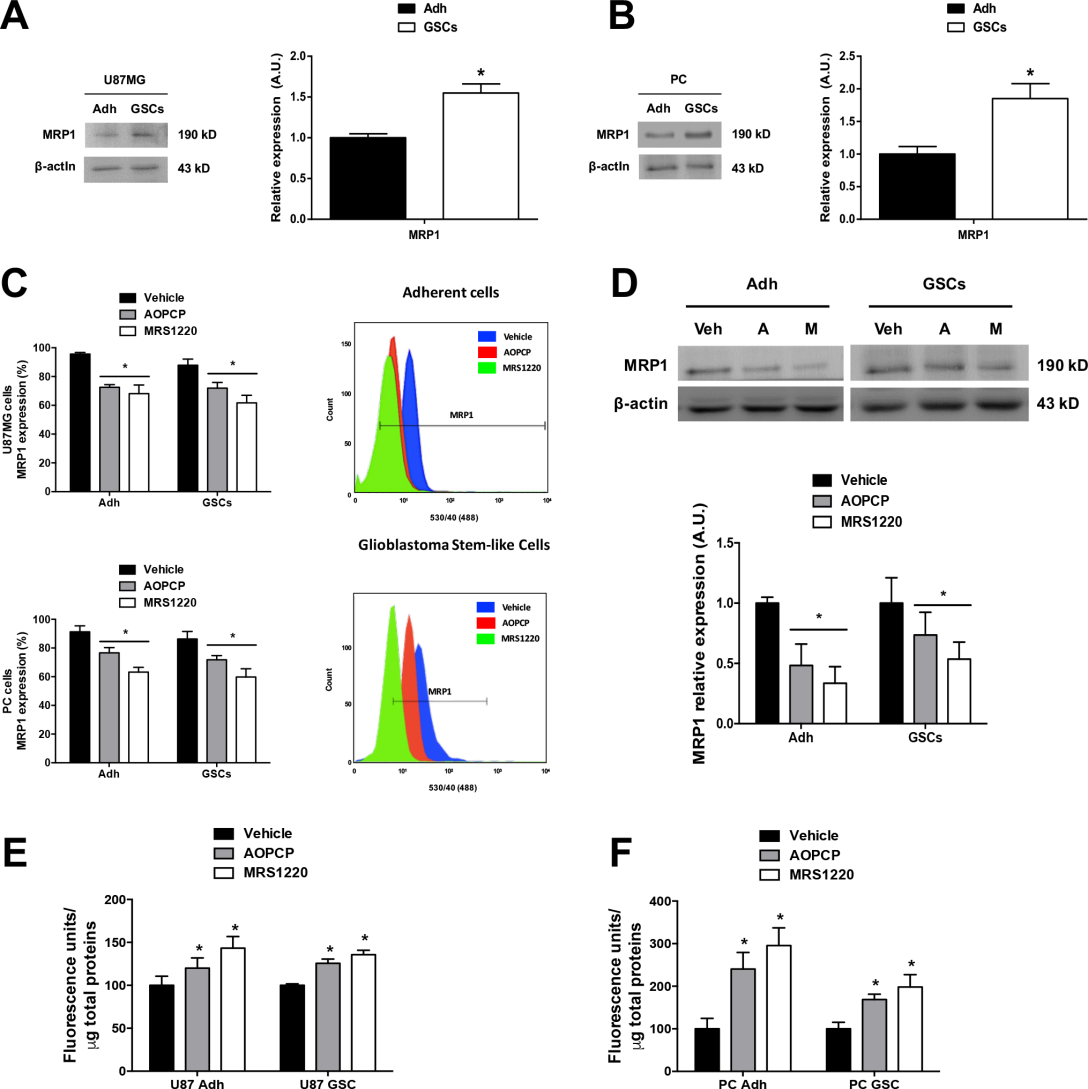
Adenosine signalling controls MRP1 transporter expression and activity in glioblastoma stem-like cells Inhibition of CD73 (AOPCP) and blockade of A_3_AR (MRS1220) decrease MRP1 transporter expression and activity in adherent cells (Adh) and GSCs in both the U87MG cell line and Primary Cultures (PC). (**A**–**B**) Western blot of MRP1 transporter in U87MG (A) and PC (B) Adh and GSCs. (**C**) Flow Cytometry graph of MRP1 transporter expression in U87MG (upper) and PC (lower) Adh and their GSCs treated with AOPCP and MRS1220 for 24 hrs. Representative flow cytometry histograms are shown (right panels) (**D**) Western blot of MRP1 transporter expression in U87MG Adh and their GSCs treated with AOPCP (“A”) and MRS1220 (“M”) for 24 hrs. (**E**–**F**) MRP1 activity in U87MG (E) and PC (F) Adh and their GSCs treated with AOPCP and MRS1220. MRP1 activity was normalized to the total protein concentration in each test. Cells treated with DMEM-0.001% DMSO (Vehicle) were used as the control condition. Graphs represent the mean ± S.D. *P < 0.05 Adh versus GSCs (A–B); *P < 0.05 versus control condition (vehicle) (C–F). *n* = 6.

In agreement with previous studies on chemoresistance in GBM specimens [5, 8, 23], the Multiple drug Resistance Protein-1 (MRP1) was detected in adherent cells; however in the present study we found that MRP1 protein and mRNA content was greater in GSCs than adherent cells of the U87MG cell line and PC cells (Figure 2A and 2B; Supplementary Figure S2). Likewise, the percentage of MRP1 transporter positive cells was greater in GSCs than adherent cells (Figure 2C).

This correlates with increased AMPase activity and A_3_AR expression levels in these cells, suggesting a link between purinergic signalling and MDR mediated by MRP1. We evaluated the effect of AOPCP (a competitive inhibitor of CD73) and MRS1220 (a selective A_3_AR antagonist) on MRP1 expression. Using flow cytometry, we observed that the porcentage of adherent cells and GSCs from the U87MG cell line and PC cells containing MRP1 was decreased with both treatments, observing a greater decrease with MRS1220 (Figure 2C). Similarly, through Western Blot analysis we observed that the treatments also decreased MRP1 protein expression in adherent cells and GSCs of U87MG cells with a more exaggerated effect observed in treatment with MRS1220, obtaining a loss of over 45% of transporter expression in GSCs (Figure 2D). In turn, we presume that these treatments would have an effect on cell chemoresistance potential. To study extrusion activity mediated by MRP1, we assessed intracellular accumulation of Carboxyfluorescein Diacetate (CFDA) in loaded cells [24]. We found that extrusion of CFDA decreased in adherent cells and GSCs upon treatment with AOPCP and MRS1220, denoted by intracellular accumulation of the fluorescent tracer in U87MG (Figure 2E) and PC cells (Figure 2F). This supports the essential role of A_3_AR in decreasing MRP1 transporter expression and activity. To validate the observed effects of A_3_AR pharmacological inhibition we also evaluated the consequence of eliminating receptor expression in U87MG cells (U87MG^KO^).

**Figure 3:**
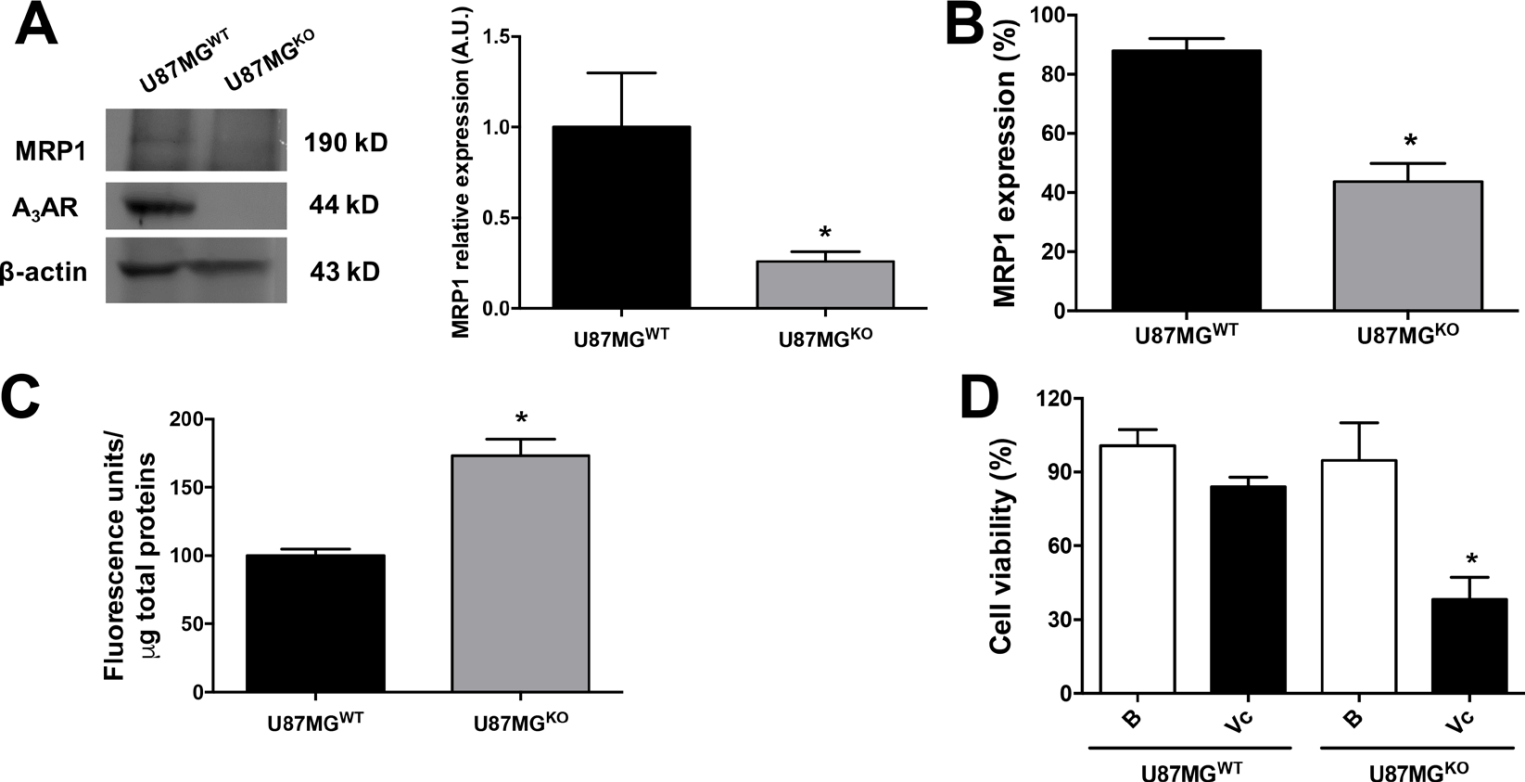
GSCs A3AR knockout show high chemosensitivity to vincristine treatment (**A**) Characterization of A_3_AR knockout in U87MG (U87MG^KO^) and U87MG wild type (U87MG^WT^) GSCs and MRP1 expression by western blot. (**B**) Graphs represent the mean ± S.D. of MRP1 expression measured by flow cytometry. (**C**) MRP1 activity in U87MG^WT^ and U87MG^KO^ GSCs. (**D**) Cell viability in human U87MG^WT^ and U87MG^KO^ GSCs treated with Vincristine (Vc; 100 nM). Cells treated with DMEM-0.001% DMSO (Vehicle) were used as the control or basal condition (B). Graphs represent the mean ± S.D. *P < 0.05 U87MG^WT^ versus U87MG^KO^ (A–C); *P < 0.05 versus control condition (D). *n* = 6.

A_3_AR expression was completely abolished using the CRISPR/Cas9 system in U87MG cells (Figure 3A and Supplementary Figure S3). Once characterized, U87MG wild type (U87MG^WT^) and U87MG^KO^ cells were cultured to generate GSCs, and then their stemness abilities and MRP1 protein content/activity were evaluated. GSCs lacking A_3_AR exhibited common properties of Cancer Stem Cells forming neurospheres clusters expressing the Stem Cells markers (CD44, CD133 and Nestin) (Supplementary Figure S4). In GSCs of U87MG^KO^ total MRP1 content was decreased (Figure 3A) and only half of the cells contained MRP1 (Figure 3B) compared to U87MG^WT^ GSCs. Consequently, intracellular accumulation of CFDA was higher in GSCs derived from U87MG^KO^ due to a lower extrusion activity mediated by MRP1 (Figure 3C). We conclude that A_3_AR signalling increases MRP1 expression and activity maintenance of MRP1 in GSCs derived from the U87MG cell line and that this receptor arises as a pharmacological target to revert the MDR phenotype in GSCs.

### A_3_AR increases MRP1 transporter expression through the PI3K/Akt and MEK/ERK1/2 signalling pathways

**Figure 4:**
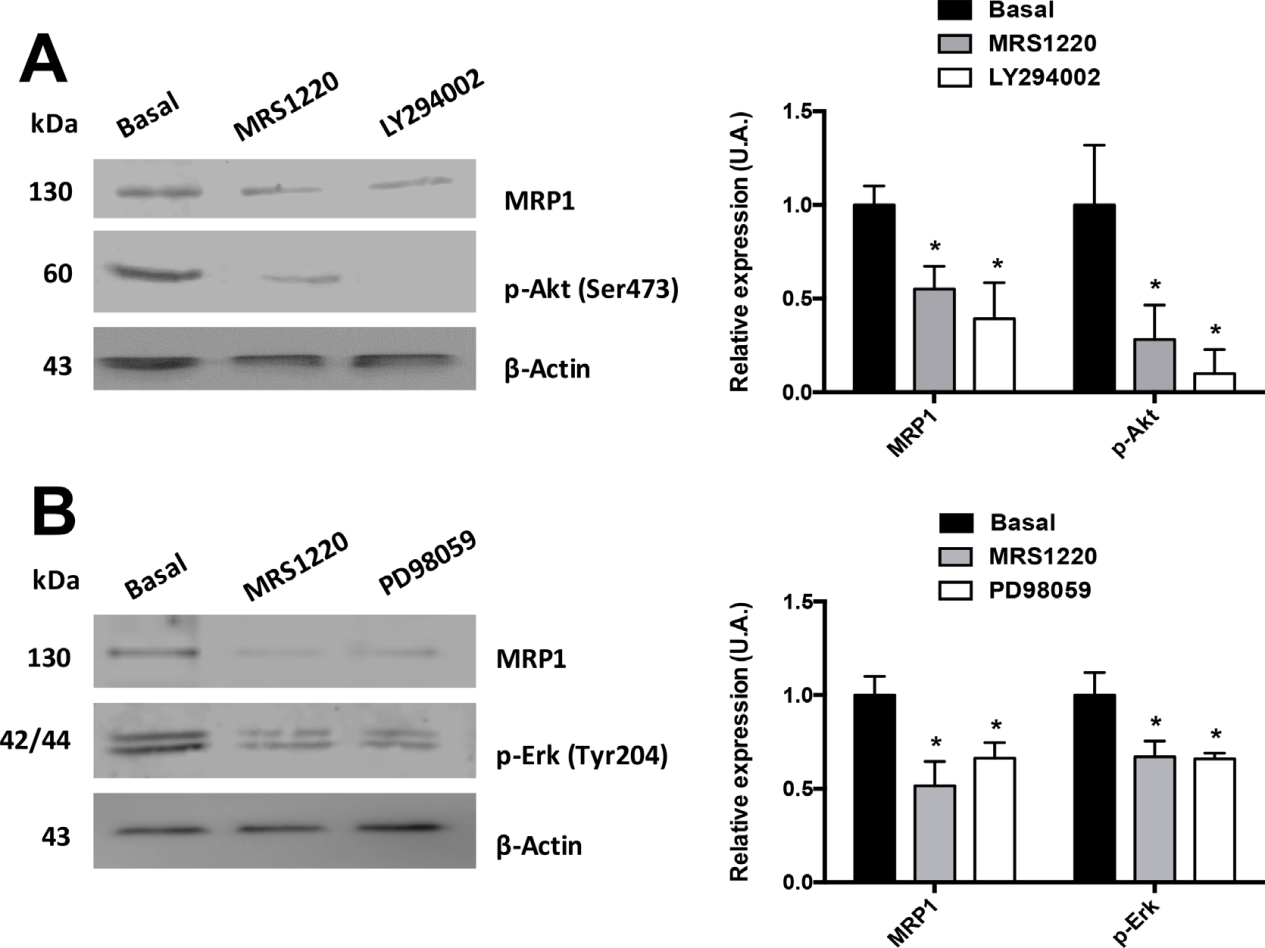
Signalling pathways involved in A_3_AR dependent MRP1 regulation MRP1 expression and activation of Akt and ERK1/2 were analysed using Western Blot. U87MG GSCs were incubated with LY294002 (50 nM), PD98059 (PD 10 μM) or MRS1220 for 24 hours. (**A**) MRP1 expression and Akt phosphorylation (Ser473) in U87MG GSCs treated with MRS1220 and LY294002. (**B**) MRP1 expression and Erk-1/2 phosphorylation (Tyr204) in U87MG GSCs treated with MRS1220 and PD98059. Cells treated with DMEM-0.001% DMSO (Vehicle) were used as the control condition (Basal). Graphs represent the mean ± S.D. *P < 0.05 versus control. *n* = 3.

Previous studies demonstrated that MRP1 transporter expression and/or activity is controlled by the PI3K/Akt and MEK/ERK1/2 signalling pathways in different tumour and CSC cell lines [25–28]. Therefore, we evaluated if A_3_AR uses these signalling pathways to regulate MRP1 in U87MG GSCs. Previously we investigated the Stemness abilities of GSCs under MRS1220, LY294002 and PD98059 treatment (Supplementary Figure S5). These treatments did not affect the neurosphere formation and stem cell markers expression (CD44 and CD133) (Supplementary Figure S5). We found that MRP1 expression was significantly decreased when U87MG GSCs were incubated with LY294002 (Figure 4A) or PD98059 (Figure 4B) for 24 hours. However, this effect was more prominent with LY294002, decreasing MRP1 expression to similar levels as treatment with MRS1220 (Figure 2D). To discover if the decreased MRP1 expression observed with LY294002 or PD98059 is induced by A_3_AR signalling, we incubated U87MG GSCs with MRS1220 for 24 hours and analysed the phosphorylation state of Akt (Figure 4A) and ERK1/2 (Figure 4B). Our results indicate that p-Akt and p-ERK1/2 expression are decreased when antagonizing A_3_AR (Figure 4). Therefore, A_3_AR activates both signalling pathways, directly affecting MRP1 expression in U87MG GSCs.

### Pharmacological blockade of A_3_AR has a chemosensitizing effect on GSCs by enhancing antitumor drug actions

**Figure 5:**
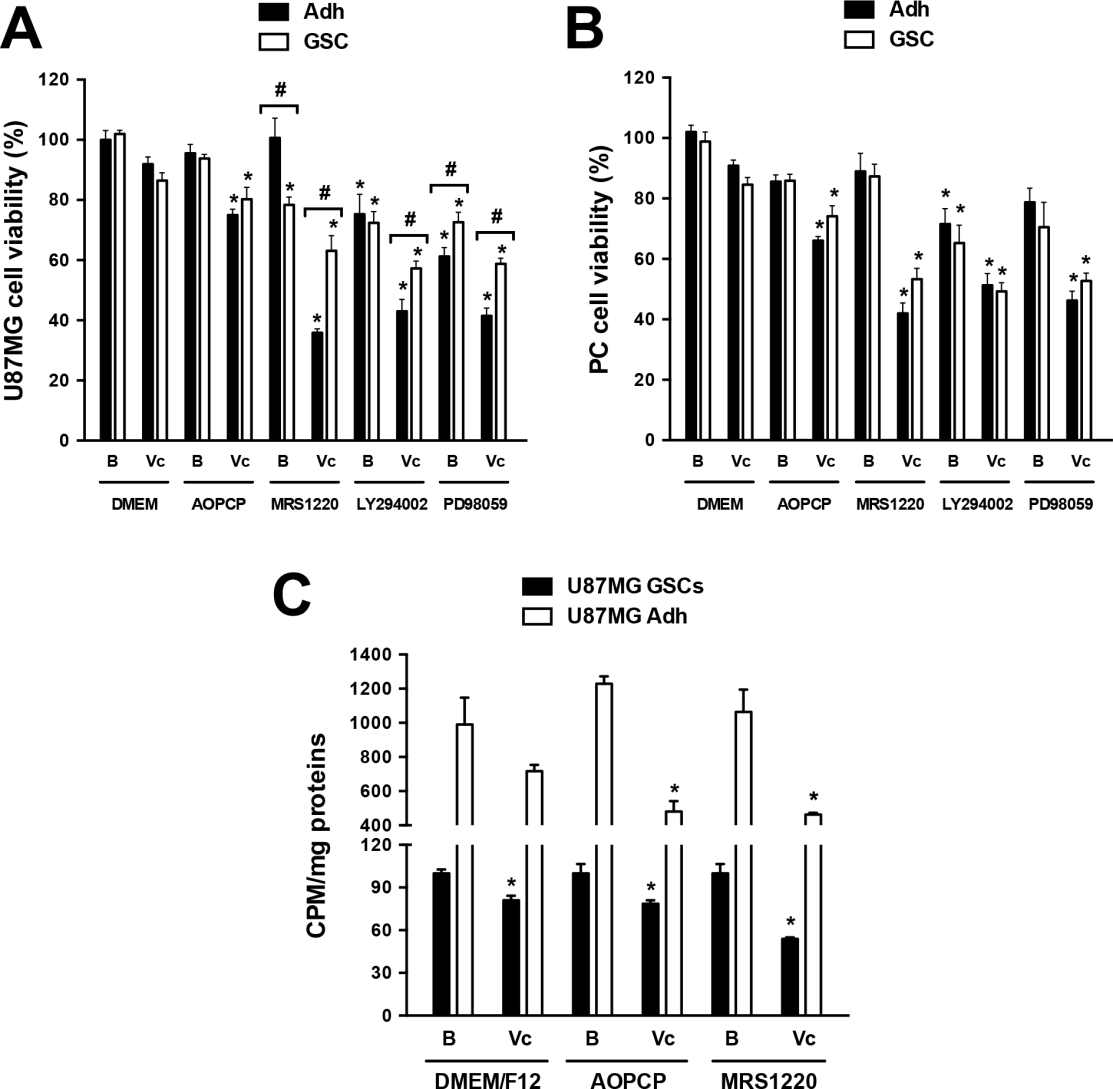
Evaluation of adenosine signalling on cell viability and proliferation of glioblastoma stem-like cells Cell viability and proliferation were evaluated by MTT and ^3^H-thymidine incorporation assays respectively. (**A**–**B**) Cell viability in U87MG (A) and PC (B) Adh cells and their respective GSCs treated with AOPCP (50 mM), MRS1220 (10 μM), LY294002 (50 nM), PD98059 (50 μM) and Vc (100 nM) for 24 hrs. (**C**) Proliferation of U87MG Adh cells and their respective GSCs treated with AOPCP (50 mM), MRS1220 (10 μM) and Vc (100 nM) for 24 hrs. Cells treated with DMEM-0.001% DMSO (Vehicle) were used as the basal (“B”) condition. *P < 0.05 versus control. ^#^*P* < 0.05 Adh versus GSCs. *n* = 5.

Further analyses in U87MG^KO^ GSCs showed that these cells were more vulnerable to the antitumor drug vincristine, a substrate to be extruded through MRP1. Over 50% of cell viability was affected by vincristine in U87MG^KO^ GSCs compared to only ~20% in U87MG^WT^ GSCs under the same treatment (Figure 3D). Pharmacological intervention of the MDR phenotype using an A_3_AR antagonist was also evaluated *in vitro*. The A_3_AR antagonist alone only slightly affected cell viability in GSCs, meanwhile MRS1220 enhanced vincristine's potential to decrease cell viability in U87MG (Figure 5A) and PC cells (Figure 5B). Moreover, the effect of LY294002 and PD98059 on the viability of U87MG (Figure 5A) and PC (Figure 5B) GSCs was evaluated. We determined that both inhibitors potentiate the effect of vincristine, being higher with LY294002 (Figure 5A and 5B). On the other hand, in adherent cells vincristine decreased cell viability when combined with MRS1220, LY294002 or PD98059 (Figure 5A and 5B). Similar effects were observed on cell proliferation, where vincristine had an antiproliferative effect on adherent cells and GSCs when used together with APOCP or MRS1220 (Figure 5C).

### Pharmacological blockage of A_3_AR has an *in vivo* chemosensitizing effect on GSCs

**Figure 6:**
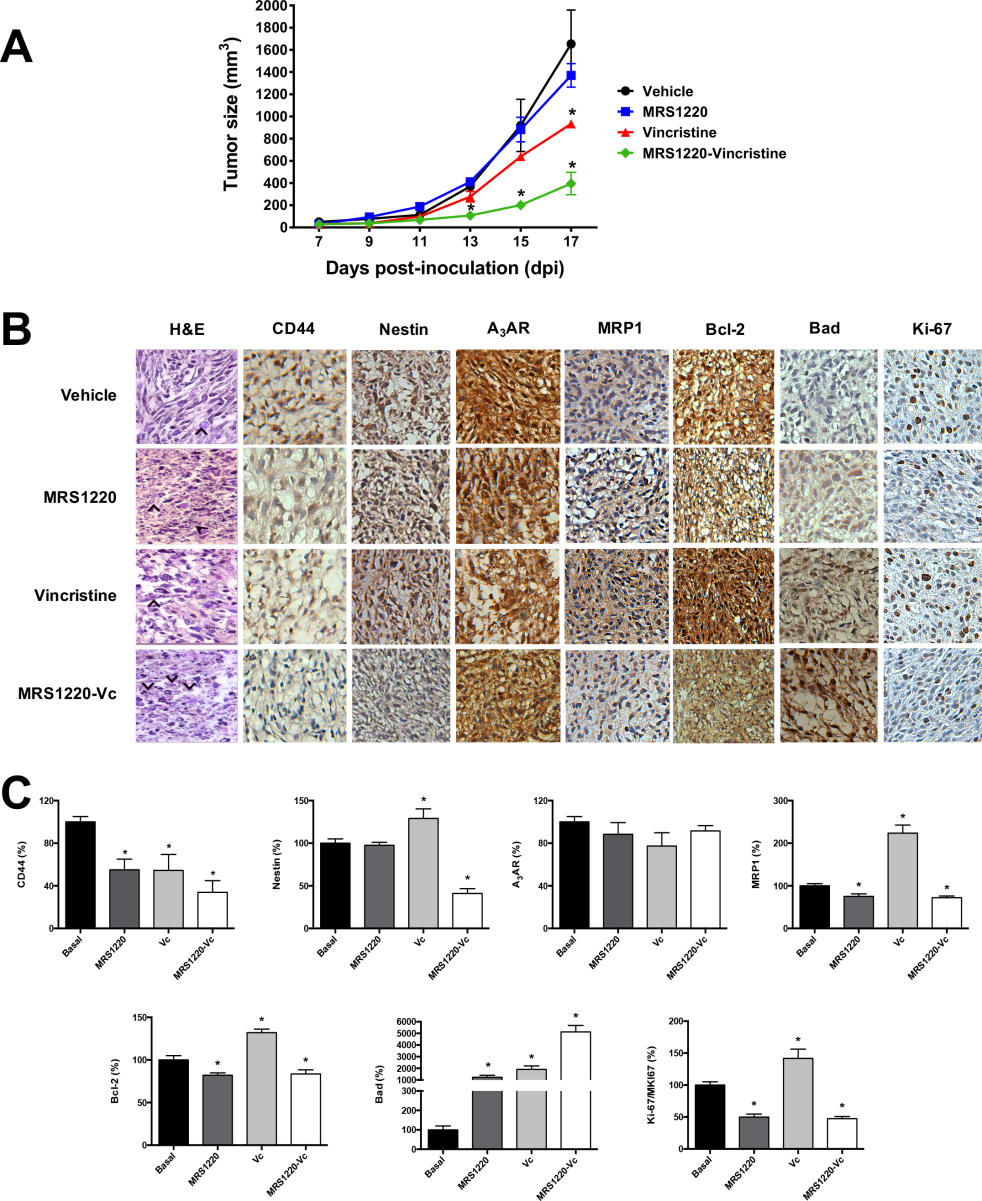
Blockade of A_3_AR has a chemosensitizing effect *in vivo* Tumours were generated by subcutaneously implanting human U87MG GSCs in NOD/SCID-IL2Rγ^null^ mice. (**A**) Graph of tumor size (mm^3^) of *in vivo* treatment. Tumours were treated following seven days post-inoculation (dpi) with 1X PBS-0.001% DMSO (Vehicle), Vincristine (Vc; 0.1 mg/kg/days), MRS1220 (0.05 mg/kg/day), and MRS1220-Vc for 10 days. (**B**) Hematoxylin & Eosin stain (H&E) and immunohistochemistry (IHC) of stem cell markers (CD44 and Nestin), A_3_AR, MRP1, Bcl-2 (anti-apoptotic), Bad, (pro-apoptotic) and ki-67 (proliferation). (**C**) IHC quantification. Tumours treated with Vehicle were used as the calibrator and normalized to 100% expression level. Graphs represent the mean ± S.D. *P < 0.05 versus control. *n* = 3.

We standardized an *in vivo* xenograft model to evaluate the effect of MRS1220 on tumours from human U87MG derived GSCs. NOD/SCID-IL2Rγ^null^ mice subcutaneously inoculated with U87MG GSCs formed a visible tumour at day 7 post-inoculation. Following tumour formation mice were treated with vincristine or MRS1220 alone or in combination. Tumour size was measured every 2 days (Figure 6A). Mice were euthanized following seventeen days of treatment and tumours were removed for histopathological analysis. Animals treated with MRS1220 alone presented a slight change in tumour growth, but this was not statistically different to vehicle treated mice. Groups treated with vincristine exhibited decreased tumour growth compared to vehicle treated mice; moreover MRS1220 notably enhanced the effect of vincristine on tumour volume (Figure 6A). Immunohistochemical analyses revealed that MRP1 staining was decreased in tumour samples from mice treated with MRS1220 (−0.25 fold), however vincristine alone increased staining (2.23 fold) (Figure 6B and 6C). Stem cells markers CD44 and Nestin were decreased in tumour samples from mice treated with vincristine combined with MRS1220, compared to the vehicle group (Figure 6B and 6C). Similar effects were observed with apoptotic markers. We observed that Bcl-2 expession (anti-apoptotic factor) decreased in tumours treated with MRS1220-Vc (17% less) and on the other hand vincristine alone increased Bcl-2 expression (Figure 6B and 6C). On the contrary, tumours from animals treated with vincristine and MRS1220-Vc presented increased Bad protein content, 19 fold and 51 fold, respectively (Figure 6B and 6C), however this effect was not observed with MRS1220 alone (Figure 6B and 6C). Finally, immunodetection of Ki-67/MKI67 (cellular proliferation marker) decreased in the tumours of mice treated with MRS1220 (50% less) and MRS1220-Vc (53% less), but not in the group treated with vincristine alone (1.42 fold) (Figure 6B and 6C). These results indicate that MRS1220 has a probed chemosensitizing effect on the anticancer drug vincristine affecting GSCs cells, responsible for giving rise to the tumour.

## DISCUSSION

Glioblastoma stem-like cells (GSCs) in GBM specimens [29] have imposed an additional challenge for GBM treatment, since they have tumorigenic capacity and are responsible for mediating relapse. Treatment success depends on the potential of GSCs to evade chemotherapy [19, 29]. The mechanisms that control MDR in these cells and their regulation are poorly understood. Our contribution in this work was to identify endogenous regulation mechanisms of MDR mediated by MRP1 in GSCs. We also demonstrated that chemosensitization of these cells interferes with A_3_AR signalling, which could be useful in early control of tumour reocurrence.

Studies in GBM demonstrate a correlation between MRP1 expression levels and a poor prognostic. MRP3 expression is associated to a low survival rate [30], however, many studies show that inhibition of MRP1 activity is sufficient to chemosensitize GBM cells to antitumoural agents that are substrates of this transporter, such as vincristine and etoposide [5, 8, 20]. GBM cells intrinsically respond to these antitumoural agents with an apparent increase in MRP1 expression. In addition to *in vitro* studies by Jin et al. (2010), our evidence from an *in vivo* tumour model shows that treatment with vincristine increases MRP1 content in tumour tissues [20]. Similar effects were observed in human samples of patients with GBM after treatment with vincristine [31]. Clinical phase studies using vincristine and etoposide, without a chemosensitizer, have not produced impact on patient survival, which reinforces the key function of MRP1 in GBM and supports the potential therapeutic use of chemosensitizers.

Endogenous regulation of MRP1 by adenosine in GSCs and the increased capacity of these cells to produce this nucleoside are new research topics. It was recently described that metabolic adaptations in GSCs can provide higher glycolytic and ATP production capacity [32, 33]. This could be a result of a higher potential to liberate ATP to the extracellular space where adenosine metabolism occurs. The microenvironment of glioblastoma cells may also contribute to increased extracellular adenosine. Low oxygen levels in the innermost areas of the tumour and cell damage caused by inflammatory processes, are two factors closely related to the increase of this nucleoside. The increased production of adenosine observed in adherent PC and PC GSCs in relation to GSCs derived from U87MG, could in fact, be the result of their response and adaptation to the tumour microenvironment. The limiting step of this pathway is AMPase activity, which was increased in GSCs compared to differentiated adherent cells. 5′-ectonucleotidase CD73 is abundant in GBM specimens, however, prostatic acid phosphatase (PAP) is present in GSCs [34]. Even though AMPase activity has not been a focus of our study, these results could explain the small inhibitory response of the CD73 inhibitor (AOPCP) on MRP1 activity, compared to the effect obtained by the A_3_AR antagonist, probably due to the existence of additional AMPase activity mediated by PAP. Therefore, the use of an A_3_AR antagonist is a more suitable therapeutic target for the inhibition of ligand production.

Under certain pathological conditions, such as hypoxia, adenosine levels can considerably increase in the extracellular fluid to the micromole range, which permits activation of low affinity adenosine receptor subtypes A_2B_AR y A_3_AR [35]. Therefore, signalling through these receptors has been associated to malignant and aggressive phenotypes in various types of cancers, such as breast cancer [36–38]. Abundance of the A_3_AR subtype has been previously reported in glioma cells [5, 39], and its activation produced pro-angiogenic effects during VEGF production and proliferation. Our *in vivo* results show that treatment with the A_3_AR antagonist decreases tumour growth. This could be explained by a possible effect of MRS1220 on GSCs and differentiated cell proliferation, as occurs *in vitro* with U87MG cells. Similar results were obtained in C6 and U138MG glioma cells, where inhibition of adenosine production reduced cell proliferation by 30% [40]. Adenosine signalling is linked to maintenance of the GSCs chemoresistant phenotype. Thus the use of MRS1220 or A_3_AR knockout sensitizes GSCs to vincristine, inhibiting tumoural growth of GSCs implanted into immunocompromised mice. Therefore, downstream intracellular signalling molecules of this adenosine receptor control MRP1 activity. Some studies reported that inhibition of PI3K/Akt and MEK/ERK1/2 pathway affect MRP1 mediated chemoresistance in cancer cells [27, 28]. Wortmannin discovered decreased phosphorylated Akt levels in myelogenous leukaemia cells, resulting in downregulation of MRP1 and decreased rho123 extrusion [25]. Also, inhibition of PI3K activity chemosensitized prostate cancer cells and MRP1 expression was inversely increased by PI3K activation in these cells [26]. Molecular and signaling profiles from glioblastoma specimens has permitted classification of these proneural, mesenchymal and proliferative tumour subtypes [41], with the latter being subdivided into classic and neural [42]. Akt signalling is activated in proliferative and mesenchymal subtypes, suggesting that they have higher chemoresistant potential induced by MRP1. In cancer stem cells less is known about the A_3_AR/PI3K/Akt axis. On the other hand, MEK inhibition in hepatocellular carcinoma decreases MRP1 and MRP3 transporter expression [28], indicating that activation of this pathway is involved in chemoresistance of pancreatic cancer cells [43]. Our contribution was to demonstrate that interception of the adenosine signalling axis can affect Akt and ERK1/2 activation and therefore decrease MRP1 expression in GSCs.

## MATERIALS AND METHODS

### Pharmacological agents

For *in vitro* studies AOPCP (50 μM; α,β-Methyleneadenosine 5′-diphosphate) was used as a CD73 inhibitor (M8386; Sigma, Saint Louis, MO) and MRS1220 (#1217; 10 μM) as a selective antagonist of A_3_AR [44]. Vincristine (Vc; 100 nM; #1257) was used as a drug substrate of the MRP1 transporter and LY294002 (LY 50 nM; #1130) as a PI3K inhibitor. All products were purchased from Tocris Biosciences, Minneapolis, MN. PD98059 (PD 50 μM; #167869, Calbiochem) was used as a MAPK inhibitor. For *in vivo* studies we used MRS1220 (0.15 mg/kg/72 hrs) and Vc (0.3 mg/kg/72 hrs) in NOD/SCID-IL2Rg^null^ mouse.

### Brain tumours

Tissue samples were obtained from surgical resection procedures of patient brain tumours at the Departamento de Neurocirugía, Instituto de Neurocirugía Asenjo, Santiago, Chile. All procedures were carried out with the approval of the Bioethics Committee of the Universidad Austral de Chile (Permit Number: 29–2011) and Servicio de Salud Metropolitano Oriente. Samples were processed for primary cultures as previously described [16, 23].

### Cell line and primary cultures

The human GBM U87MG cell line was acquired from the American Type Culture Collection Company (ATCC^®^ HTB-14™). Cells were grown in DMEM-F12 medium supplemented with 10% fetal bovine serum, 100 U/mL penicillin and 100 μg/mL streptomycin, all purchased from Life Technologies. Cells were incubated in standard conditions at 37°C in a humidified atmosphere with 5% CO_2_. For preparation of GBM Primary Cultures (PC) [16, 23] the resected tissues were washed twice with HBSS-1× antibiotic-antimitotic (Life Technologies) in aseptic conditions. After removing visible blood vessels and necrotic tissues, glioblastoma samples were centrifuged (600 g by 5 min) and the sediment was incubated by 30 min with 6 mg/mL of collagenase type I (Gibco^®^) at 37°C in a shaker platform. The digested tissue was filter through a nylon membrane (40 μm) and centrifuged (600 g by 5 min). Sediment was resuspended in DMEM/F-12 supplemented with 20% fetal bovine serum, 100 units/mL of penicillin/streptomycin and incubated at 37°C in a humidified atmosphere and 5% CO_2_. To form neurospheres (GSCs) cells were grown in Neurobasal medium (Gibco^®^) supplemented with EGF (20 ng/mL), bFGF (20 ng/mL), LIF (10 ng/mL), 1 × B27 (w/o vitamin A) and 100 units/mL of penicillin/streptomycin.

### Immunocytochemistry

Adherent human Primary Cultures (PC) and U87MG cell lines were grown on circular coverslips at semi-confluence, and GSCs neurospheres were adhered to coverslips using Poly-L-Lysine Cell Attachment Protocol [45]. Later both kinds of cells were washed with 0.1 M phosphate buffer (pH 7.4), fixed with paraformaldehyde 3.7% and permeabilized using 0.3% Triton X-100 in 1 × PBS. Preparations were blocked with 1% BSA and incubated with anti-CD44 (ab51037, abcam), anti-Nestin (ab22035, abcam), anti-GFAP (sc-33673, Santa Cruz), and anti-TUBB3 (SAB4700544, Sigma Aldrich) antibodies. For Immunocytochemistry the ImmPRESS HRP Universal Antibody Polymer Detection Kit (Vector Laboratories) and Liquid DAB+Substrate Chromogen System (K3468, DAKO) were used. Finally, hematoxylin staining was used as a counter stain and cells were visualized using a light microscope (Zeiss).

### Protein extracts and Western blots

Total protein extracts of differentiated cells (called adherent cells or Adh) and GSCs from the U87MG cell line were obtained by lysis with 150 μL extraction buffer (63.5 mM Tris/HCl pH 6.8, 10% glicerol, 2% SDS, 1 mM PMSF, 2 mM aprotinin, 1 mg/mL leupeptin and pepstatin). Samples were sonicated three times for 5 sec and centrifuged at 12.000 g for 15 min, then protein concentration was quantified using Bicinchoninic Acid (BCA) Protein Assay (BioRad). For Western blots, 50 μg of protein extract was fractionated by SDS-PAGE and transferred to 0.22 μm PVDF membranes (PERK1/2in–Elmer). Membranes were blocked in 1 × PBS/0.05%tween/1%BSA and incubated with anti-CD44 (562991, BD Bioscience), anti-CD133 (130-092-395, Miltenyi Biotec), anti-Nestin (sc-33677, Santa Cruz), anti-GFAP (sc-33673, Santa Cruz), anti-TUBB3 (SAB4700544, Sigma Aldrich), anti-MRP1 (sc-18835, Santa Cruz), anti-A_3_AR (sc-13938, Santa Cruz), anti-pAkt (S473, 9271, Cell Signalling), and anti-pERK1/2 (sc-7383, Santa Cruz) primary antibodies. A secondary antibody-HRP conjugate was used for immunodetection and was visualised using the ECL Western blotting chemiluminescence system (Amersham Pharmacia Biotech, Durham, NC). Finally images were captured with the Syngene G:Box system (Synoptics Ltd) and were quantified using a densitometry analysis with ImageJ software (NIH). Each membrane was normalized to β-actin (sc-47778 HRP, Santa Cruz) expression.

### Flow cytometry analysis

Differentiated cells (adherent cells) and GSCs of the U87MG cell line were evaluated by flow cytometry (BD C6 Accuri and BD FACSJazz™, USA). Briefly, 1.0 × 10^6^ cells were fixed with PFA 3.7% for 15 min at room temperature and permeabilized with cold methanol for 45 min at 4°C. Cells were then blocked for 45 min with 1 × PBS-BSA 0.5% at room temperature. Lastly, the cells were marked with the anti-MRP1 (sc-18835, Santa Cruz), anti-A_3_AR (sc-13938, Santa Cruz), anti-Nestin (sc-23927, Santa Cruz), and anti-CD133 (130-092-395, Miltenyi Biotec) antibodies at 4°C overnight and were detected by flow cytometry using their respective Alexa fluor 488 antibody (Life Technology) through the FL1 filter (530/30 nm) of the cytometer.

### RT-qPCR

We used an RT-qPCR assay, with the ΔΔCt method and ACTB as a normalizer gene, to evaluate mRNA expression of Stem cell markers (CD44, Nestin and CD133) and MRP1 in PC and U87MG cell line. Reverse transcription was performed with 1 μg of RNA using the tetro cDNA Synthesis kit (Bioline^®^). The qPCR reaction was performed in a final volume of 20 μL with 250 nM of each primer using the 5× HOT FIREPol^®^ EvaGreen^®^ qPCR Mix Plus (ROX) kit (Solis BioDyne, Tartu, Estonia) following the manufacturer›s instructions. Briefly, HOT FIREPol^®^ DNA Polymerase was activated by a 15 min incubation step at 95°C, followed by 40 cycles of denaturation (95°C by 15 sec), annealing (60°C by 20 sec) and elongation (72°C by 20 sec). Finally, a dissociation curve was assayed to distinguish specific amplicons. All qPCRs were conducted in triplicate for each treatment. GraphPad Prism^®^ 6.01 software was used to graph the results and calculate statistics. *P value* < 0.05 was considered statistically significant. The list of primers used in this study was added to the supplementary information Table Supplementary S1.

### Adenosine quantification

Cells (Adherent cells and GSCs) from PC and U87MG were incubated in 1 mL of Tyrode's buffer for 1 h at 37°C. A volume of 200 μL of incubation medium was mixed with 100 μl of citrate buffer at pH 6.0. Adenosine, AMP, ADP and ATP contents were quantified with 2-chloroacetaldehyde derivatizations by HPLC fractionation in a Chromolith Performance RP-18 column (Merck) and by fluorescent detection [46]. Quantification procedures included adenosine (A9251), ATP (FLAAS), ADP (A2754) and AMP (01930) standards all purchased from Sigma Aldrich. Concentration was normalized to the total protein concentration in each test. For CD73 activity, adherent cells and GSCs of PC and the U87MG cell line (2 × 10^5^) were exposed to AMP (400 nM) for 15 min in Tyrode's buffer at 37°C and at controlled oxygen levels. The final adenosine content was quantified and normalized as described above.

### MRP1 functional assays

For MRP1 functional assays we used the protocol described by Garrido et al. (2011) [24]. Human PC and U87MG adherent cells with their respective GSCs (2 × 10^5^) were exposed to CD73 activity inhibitor (AOPCP) or an A_3_AR antagonist (MRS1220) in serum free medium DMEM/F-12 for 24 h at 37°C in 24-well plates. They were then washed and loaded with 500 nM of the fluorescent substrate CFDA for 15 min. Cells were subsequently washed three times with 1 × PBS and incubated for 15 min in serum-free DMEM/F-12 medium. Cells were then washed three times with ice-cold 1 × PBS and lysed in 1 × PBS containing 0.4% Triton X-100. Fluorescence of cell extracts was measured using a spectrofluorometer (PERK1/2in–Elmer), exciting at 488 nm and collecting emission at 530 nm [47]. Accumulated fluorescence was corrected to the total protein content of lysed cells.

### Cell viability assay

For cell viability assays the MTT protocol described by Garrido et al. (2011) [24] was performed. A quantity of 10 × 10^4^ cells per well were cultured in 96-well plates for 24 h and exposed to Vc (100 nM) or Et (2 μM) alone or in combination with a CD73 inhibitor (AOPCP), A_3_AR antagonist (MRS1220), PI3K inhibitor (LY294002) or a MAPK inhibitor (PD98059) for 24 h. Cells were then incubated with 5 mg/ml MTT reagent (thiazole blue tetrazolium) in culture medium for 1 h. Formazan crystals were dissolved in 100 μl of DMSO. Some modifications were made for the neurosphere MTT assay. Briefly, GSCs after seven days of culture were seeded in 96-well plates for 24 h using the treatment previously described. GSCs were then incubated with MTT reagent (5 mg/ml) for 4 h, formazan crystals were dissolved in MTT lysis buffer (100 μl of 10% SDS, 45% dimethylformamide and pH was adjusted to pH 4.5 with glacial acid) overnight at 37°C. For both assays absorbance was measured at 550 nm using a microplate reader (Synergy HT, BioTek Instruments, Inc.). Absorbance values were expressed as a percentage relative to control cells without treatment (basal condition).

### Cell proliferation assay

The proliferative response was evaluated in human U87MG cell line by ^3^H-thymidine incorporation assay [24]. Briefly, 1 × 10^6^ cells per well were cultured in 12-well plates and incubated with Vc (100 nM) and Et (2 μM) alone or with CD73 inhibitor (AOPCP) or A_3_AR antagonist (MRS1220) for 24 h. Then 0.5 μCi/mL of ^3^H-thymidine per well was added for 24 h. After incubation, cells were harvested and the resulting trichloroacetic acid-insoluble materials were collected on 0.45 μm PVDF filters. Once dry, the filters were put into plastic vials with 4 mL of liquid scintillation. Incorporated radioactivity was measured in a liquid scintillation counter (Packard). The results were plotted as counts per minute (cpm) and normalized to the total protein content of each culture well.

### Generation of A_3_AR knockout GSCs

For the generation of a human U87MG A_3_AR Knockout (U87MG^KO^) cells we used the CRISPR-Cas9 system [48]. The plasmidial vector contained the Cas9 endonuclease and gRNA was purchased at Santa Cruz Biotechnology (sc-402007). We generated stable U87MG^KO^ cells, by homology directed repair (HDR), that were selected by the expression of RFP and puromycin resistance (sc-4020078-HDR) [49]. To validate U87MG^KO^ cells we used western blot and flow cytometry. Finally, these cells were cultured as GSCs and we then evaluated expression (western blot and flow cytometry) and activity (CFDA assay) of MRP1, and cell viability (MTT assay) under the previously described treatment.

### Animals and treatment

All animal studies were approved by the Institutional Animal Care and Use committee at the Universidad Austral de Chile (Permit Number: 29–2011) according to the NIH Guide for the Care and Use of Laboratory Animals. NOD/SCID-IL2Rγ^null^ mice (No.005557; The Jackson Laboratory^©^, USA) weighing 25 g were maintained under standard laboratory conditions: 12 h light-dark cycle (lights on from 7:00 a.m. to 7:00 p.m.), with food and water ad libitum. Human U87MG GSCs were grown, collected and resuspended at 1 × 10^5^ cells/200 μl (DMEM/F12). Cells were inoculated by subcutaneous injection at the left flank of mice previously anesthetized via intraperitoneal administration of ketamine (100 mg/kg)/xylazine (10 mg/kg). 7 days post-inoculation, mice were treated with vehicle (DMEM:F12-0.001% DMSO), MRS1220 (0.05 mg/kg/day), Vincristine (Vc; 0.1 mg/kg/days) or MRS1220-Vc for 10 days. Tumour growth was measure daily and plotted (cm^3^). Following 10 days of treatment mice were euthanized by intraperitoneal administration of Sodium Thiopental (120 mg/kg). Subcutaneous tumours were removed and fixed in 3.7% paraformaldehyde (Sigma-Aldrich, Germany).

### Histopathology and immunohystochemistry

Tumour samples were processed at the Pathological Anatomy Department of the Hospital Base of Valdivia, where tumour samples were paraffin embedded and 5 μm sections were mounted on silanized slides. Histological preparations were dewaxed with xylol and rehydrated using alcohols in decreasing concentration. For histopathology analysis samples were immersed in haematoxylin for 5 min and washed preparations were immersed with 0.1 M sodium borate (pH 8.5) for 30 sec and eosin for 5 min. Finally, samples were passed through another series of alcohols in ascending order from 70° to 100° followed by xylol and mounted (Histomount, Invitrogen). The preparations were visualized using a light microscope (Zeiss). For immunodetection samples were treated as described previously [43]. Briefly, samples were dewaxed and hydrated using alcohols in decreasing concentration, immersed in citrate buffer (pH 6) and heated until boiling. 3% hydrogen peroxide was added to the samples and washed with distilled water and 1 × PBS. Preparations were blocked with 5% BSA for 1 h and incubated with anti-MRP1 (sc-18836; Santa Cruz), anti-A_3_AR (sc-13938, Santa Cruz), anti-Ki67 (sc-15402; Santa Cruz), for Stem Cells [anti-Nestin (ab22035, abcam), and anti-CD44 (ab51037, abcam)] and apoptotic [anti-Bcl-2 (610538) and anti-Bad (610391) antibodies, all purchased from BD Biosciences] markers. Immunodetections were visualised using the ImmPRESS HRP Universal Antibody Polymer Detection Kit (Vector Laboratories) and Liquid DAB + Substrate Chromogen System (K3468, DAKO) following the manufacturer's instructions. The distribution of markers in the tissue was visualized using a light microscope (Zeiss) and a representative region of each condition (Control, Vincristine, MRS1220 and MRS1220-Vincrsitine) was used to evaluate expression of these markers. Finally, data were processed using ImageJ software (NIH) and the GraphPad Prism^®^ 6.01 software was used to graph the results and statistics. Control values without treatment were normalized and calibrated to 100%. *P value* ≤ 0.05 was considered statistically significant.

### Statistics

Values are means ± SD, where n indicates the number of animals used or replicates in cell experiments. Statistical analysis was carried out on raw data using the Peritz F multiple means comparison test. Student's *t-test* was applied for unpaired data. *P value* < 0.05 was considered statistically significant.

## SUPPLEMENTARY MATERIAL FIGURES AND TABLE


