# Supplementary material for: Adenosine A3 receptor elicits chemoresistance mediated by multiple resistance-associated protein-1 in human glioblastoma stem-like cells
Source: Oncotarget. 2016 Sep 15;7(41):67373–86. doi: 10.18632/oncotarget.12033 (PMC5341882; doi:10.18632/oncotarget.12033)
Supplement: Supplementary file 1 [file oncotarget-07-67373-s001.pdf]

Adenosine A_3 receptor elicits chemoresistance mediated by multiple resistance-associated protein-1 in human glioblastoma stem-like cells

Supplementary Materials

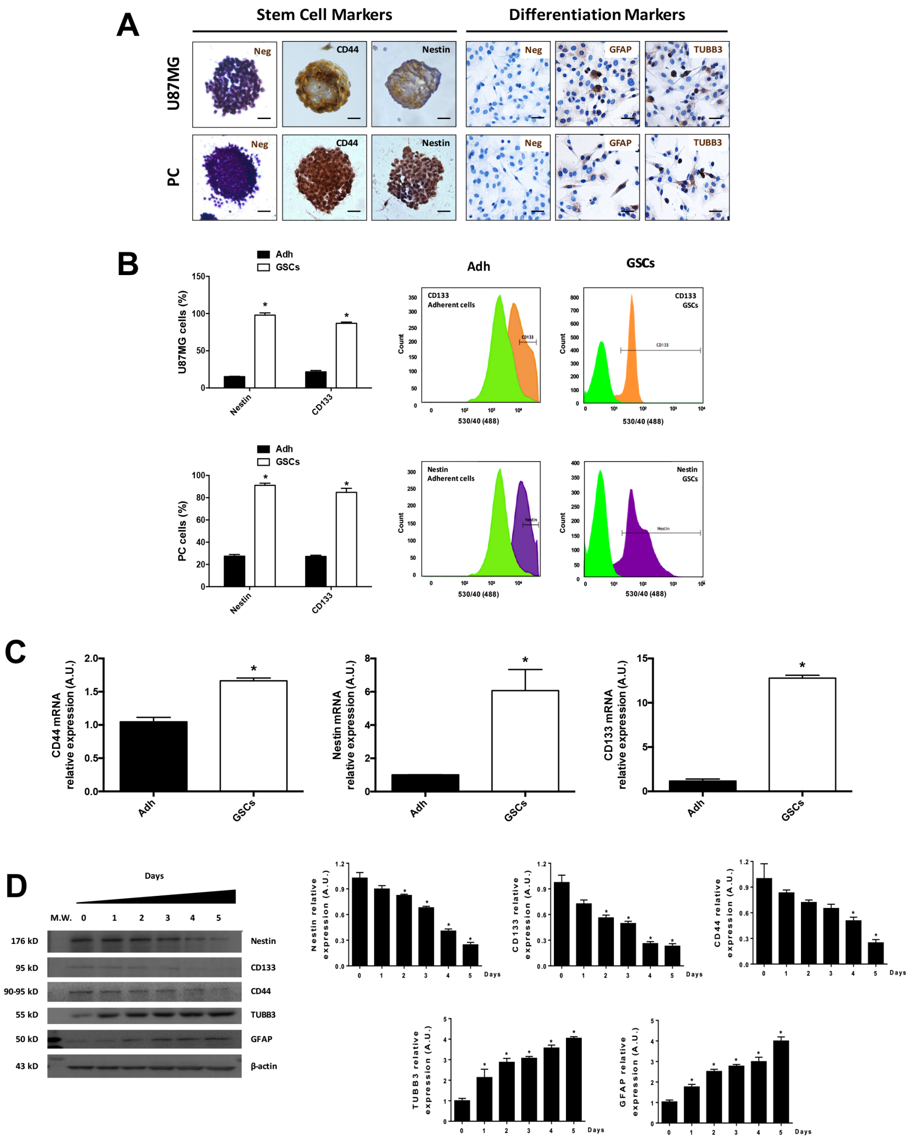

Supplementary Figure S1: Characterization of glioblastoma stem-like cells. The human U87MG cell line and Primary Cultures (PC) of human GBM form aggregates of Glioblastoma Stem-like Cells (GSCs) called neurospheres that express Stem Cell markers. (**A**) Immunocytochemistry analysis of U87MG and PC GSCs expressing Stem Cell markers (CD44 and Nestin) at day seven of culture in neurosphere medium (neurobasal medium plus growth factors). These cells when exposed to culture medium with serum grow as adherent (Adh) cells and express differentiation markers of glial cells (GFAP) and neurons (TUBB3) at day five of culture. (**B**) Flow cytometry of Stem Cell markers (Nestin and CD133) in U87MG and PC adherent cells and GSCs (left panels). Representative flow cytometry histogram are presents (right panels) (**C**) mRNA expression of Stem cell markers (CD44, Nestin and CD133) by RT-qPCR in U87MG Adh cells and GSCs. (**D**) Stem cell (Nestin, CD133 and CD44) and differentiation markers (GFAP and TUBB3) evaluated by Western blot in U87MG GSCs exposed to culture medium with 10% Fetal Bovine Serum for five days. U87MG GSCs at day zero were normalized to 1. Graphs represent the mean ± S.D. *$P < 0.05$ Adh versus GSCs (B–C); *$P < 0.05$ versus day zero (D). $n = 6$.

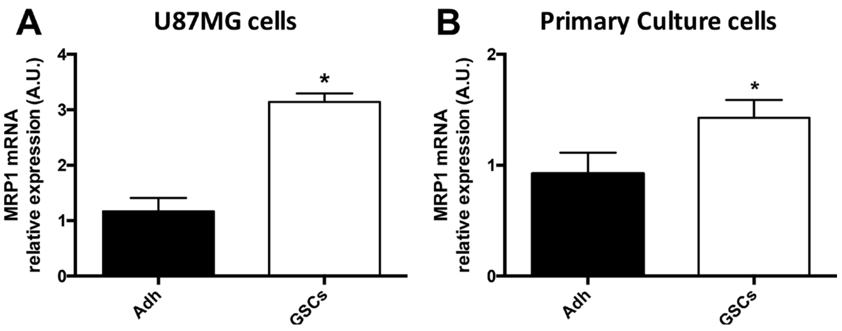

Supplementary Figure S2: High mRNA expression of MRP1 transporter in glioblastoma stem-like cells. mRNA expression of MRP1 by RT-qPCR in human U87MG and PC adherent (Adh) cells and GSCs. Graphs represent the mean ± S.D. **P* < 0.05 Adh versus GSCs. *n* = 6.

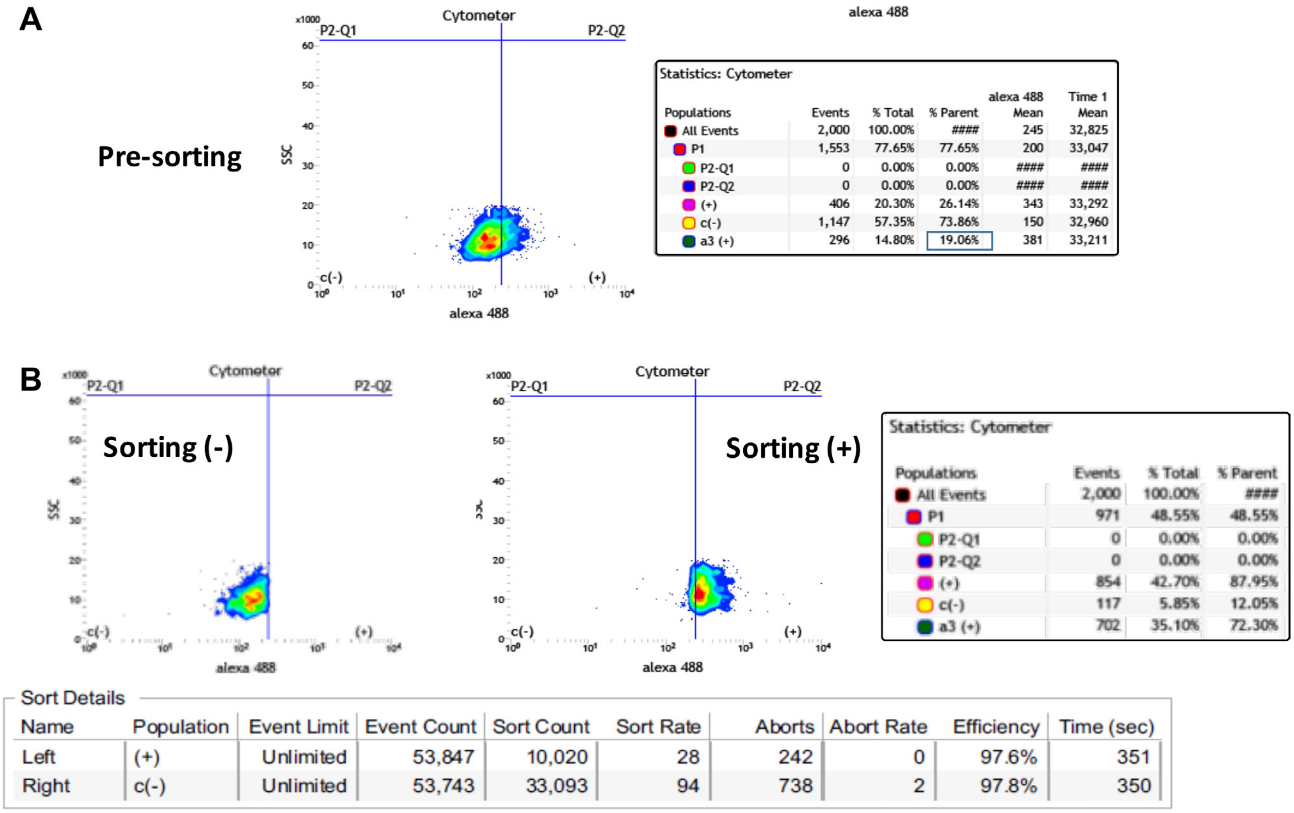

Supplementary Figure S3: Characterization and sorting of U87MG knockout A_3AR cells by flow cytometry. (**A**) Graph represents flow cytometry (Pre-sorting) analysis of U87MG^{KO} GSCs at day fifteen post transfection with the CRISPRs/Cas9 system. Knockout generation efficiency at day fifteen was 81%. (**B**) Cell sorting of U87MG^{KO} GSCs had an efficiency of 97.8%.

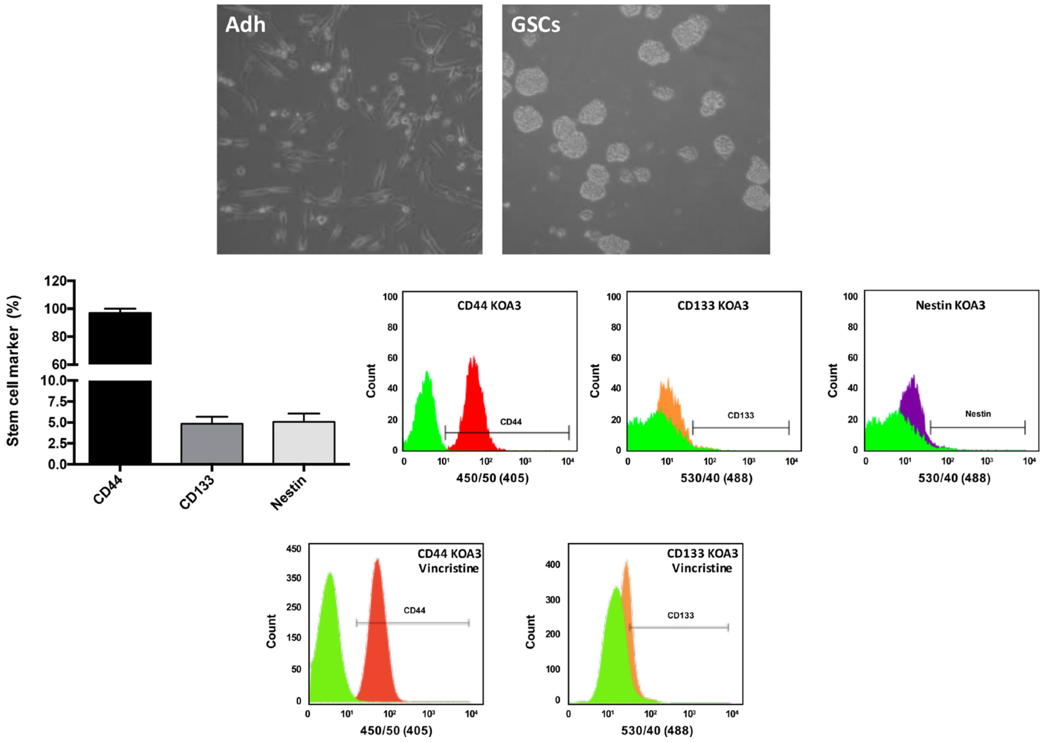

Supplementary Figure S4: Stemness abilities of U87MG knockout A_3AR cells. The human U87MG Knockout A_3AR cells grow as adherent (Adh) cells and neurospheres (GSCs). GSCs express Stem Cell markers (CD44, CD133 and Nestin) by Flow cytometry (lower panel). Representative flow cytometry histograms are shown. Graphs represent the mean ± S.D.

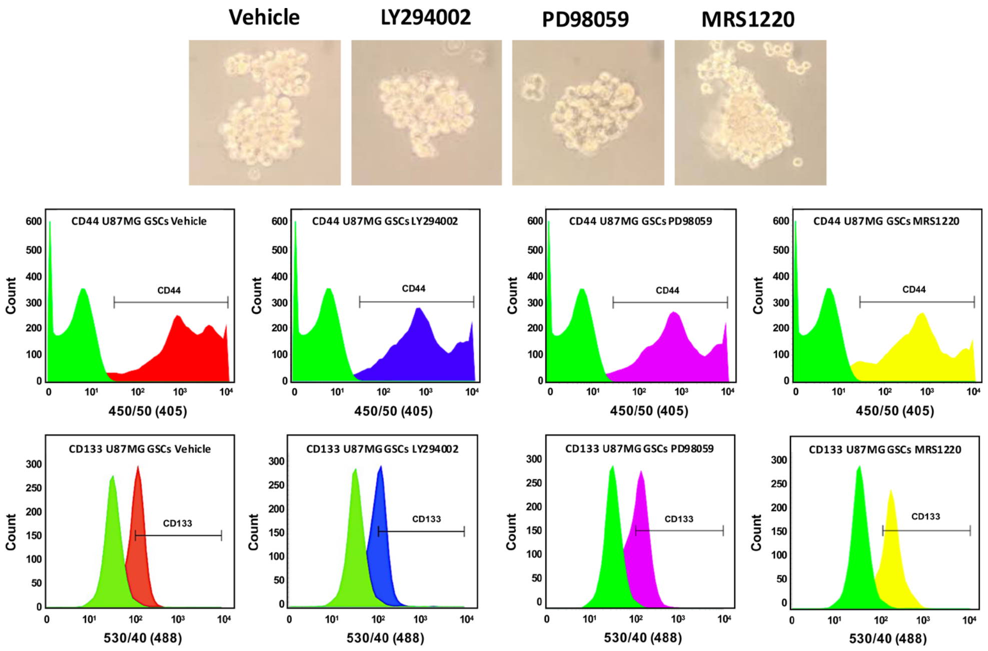

Supplementary Figure S5: Stemness abilities of U87MG GSCs under treatment with LY294002, PD98059 and MRS1220. GSCs of U87MG cells were cultured by seven days and treated with LY294002 (as a PI3K inhibitor), PD98059 (as a MAPK inhibitor) and MRS1220 (as a selective antagonist of A_3AR) by 24 hrs. U87MG GSCs treated form neurosphere clusters (upper panels) and express Stem Cell markers (CD44 and CD133) by Flow cytometry (lower panels). Representative flow cytometry histogram are shown.

Supplementary Table S1: List of primers used for RT-qPCR

Gene	Forward primer	Reverse primer
Nestin	5′-ttgcctgctacccttgagac-3′	5′-gggctctgatctctgcatctac-3′
CD133	5′-gcatgcaaaagccatcatag-3′	5′-gggaatgcctacatctggaa-3′
CD44	5′-gcccaatgcctttgatggacc-3′	5′-gcagggattctgtctgtgctg-3′
MRP1	5′-ggactttcgtgtgctcctga-3′	5′-Aggtcaagctttccgtgtactg-3′
